# A highly conserved NB-LRR encoding gene cluster effective against *Setosphaeria turcica *in sorghum

**DOI:** 10.1186/1471-2229-11-151

**Published:** 2011-11-03

**Authors:** Tom Martin, Moses Biruma, Ingela Fridborg, Patrick Okori, Christina Dixelius

**Affiliations:** 1 SLU, Uppsala Biocenter, Dept. Plant Biology and Forest Genetics, Uppsala P.O. Box 7080, S-750 07, Uppsala, Sweden; 2 Dept. of Crop Science, Makerere University P.O. Box 7062, Kampala, Uganda; 3 National Agriculture Research Organisation, P.O. Box 295, Entebbe, Uganda

## Abstract

**Background:**

The fungal pathogen *Setosphaeria turcica *causes turcicum or northern leaf blight disease on maize, sorghum and related grasses. A prevalent foliar disease found worldwide where the two host crops, maize and sorghum are grown. The aim of the present study was to find genes controlling the host defense response to this devastating plant pathogen. A cDNA-AFLP approach was taken to identify candidate sequences, which functions were further validated via virus induced gene silencing (VIGS), and real-time PCR analysis. Phylogenetic analysis was performed to address evolutionary events.

**Results:**

cDNA-AFLP analysis was run on susceptible and resistant sorghum and maize genotypes to identify resistance-related sequences. One CC-NB-LRR encoding gene *GRMZM2G005347 *was found among the up-regulated maize transcripts after fungal challenge. The new plant resistance gene was designated as *St *referring to *S. turcica*. Genome sequence comparison revealed that the CC-NB-LRR encoding *St *genes are located on chromosome 2 in maize, and on chromosome 5 in sorghum. The six *St *sorghum genes reside in three pairs in one locus. When the sorghum *St *genes were silenced via VIGS, the resistance was clearly compromised, an observation that was supported by real-time PCR. Database searches and phylogenetic analysis suggest that the *St *genes have a common ancestor present before the grass subfamily split 50-70 million years ago. Today, 6 genes are present in sorghum, 9 in rice and foxtail millet, respectively, 3 in maize and 4 in *Brachypodium distachyon*. The *St *gene homologs have all highly conserved sequences, and commonly reside as gene pairs in the grass genomes.

**Conclusions:**

Resistance genes to *S. turcica*, with a CC-NB-LRR protein domain architecture, have been found in maize and sorghum. VIGS analysis revealed their importance in the surveillance to *S. turcica *in sorghum. The *St *genes are highly conserved in sorghum, rice, foxtail millet, maize and Brachypodium, suggesting an essential evolutionary function.

## Background

The immune system has developed in a stepwise manner by progressive sophistication of basic functions that helped ancestral organisms to survive in their hostile environment. Recognition of pathogens in a species-specific way results in the generation of a very robust mode of surveillance system in plants. This form of protection termed resistance (R) protein-mediated or effector-triggered immunity is induced when a plant encoded R protein "perceives" the presence of a pathogen-derived effector molecule, represented by specific avirulence (Avr) gene products [1]. Following recognition of the pathogen, one or more signal transduction pathways are induced in the host plant and these lead to the prevention of colonization by the pathogen.

The majority of characterized R proteins encode a nucleotide-binding site (NB) and leucine-rich repeats (LRR). NB-LRR-encoding genes make up one of the largest and most variable gene families found in plants, with most plant genomes containing several hundred family members [2-6]. The N-terminal ends of R-proteins are predominantly composed of a TIR (Toll/Interleukin-1 Receptor) homologous domain or form a coiled-coil (CC) motif. Monocots in particular, have numerous CC-NB-LRR proteins in their genomes. Accumulating data suggest furthermore that N termini of R-proteins may interact with a range of pathogen-derived proteins. However, the LRR domain may determine the final outcome of this recognition, leading to downstream signaling and initiation of defense responses [7].

Many *R*-genes are located in clusters that either comprise several copies of homologous sequences arising from a single gene family or co-localized *R*-gene sequences derived from unrelated gene families [8,9]. This genomic make-up assists multiple proteins to become modified via various genic and intergenic processes enabling rapid evolution and adaptation to changes in a pathogen genome [10]. *R*-genes can also act in pairs [11,12]. The *R*-gene pairs can differ in genomic location and protein domain structure but also to their interaction with different pathogen isolates.

The heterothallic ascomycete *Setosphaeria turcica *(Luttrell) Leonard & Suggs (anamorph: *Exserohlium turcicum*, former *Helminthosporium turcicum*) causes turcicum or northern leaf blight disease on maize. This fungal pathogen also attacks sorghum and related grass species, for example Johnson grass [13,14]. Turcicum leaf blight is one of the most prevalent foliar diseases in most maize-growing regions of the world. The disease causes periodic epidemics associated with significant yield losses, particularly under conditions of moderate temperature and high humidity [15-17]. Resistance to *S. turcica *has mainly been characterized in maize. *S. turcica *was earlier named *Helminthosporium turcicum *and resistance has hitherto been designated *Ht *and conferred by major race-specific genes (*Ht1*, *Ht2, Ht3 *or *HtN*) or via partial resistance, reviewed by Welz and Geiger [18]. In our work we designate the new resistance genes as *St *referring to *Setosphaeria turcica*.

Maize and sorghum are the most important staple cereals for sub-Saharan Africa (SSA). While maize is an introduced crop [19], sorghum is believed to have been domesticated in SSA particularly in the Nile basin or Ethiopia, as recently as 1000 BC [20]. Sorghum like many other crop species experience large problems with plant pathogens, particularly fungal diseases. Turcicum leaf blight incited by *S. turcica *is one main problem [21]. This disease has been considered as of minor importance in Uganda until 1988 when it caused extensive yield losses on maize [22]. By introducing improved resistance in new varieties the threat posed by the disease was subsequently reduced. Severe and sporadic outbreaks of turcicum leaf blight have now reappeared in East Africa [23-25]. A change in the *S. turcica *population has been suggested to be the main cause of this shift in disease pattern. In order to detect potential new changes of the *S. turcica *pathogen and the turcicum leaf blight disease, a survey was undertaken in Uganda to examine the sorghum - *S. turcica *pathosystem in terms of disease severity and incidence, race patterns and new resistant resources [26]. It can be concluded from those studies that fungal isolates from sorghum could infect maize. Upon cross inoculation on maize differential lines harboring different *Ht *genes, four *S. turcica *isolates were identified as race 1, two as race 2, and one isolate corresponded to race 0 and race 3, respectively, whereas 10 isolates were unclassified. Highly resistant sorghum accessions originating from a regional collection were also identified.

In this work, we used cDNA-amplified fragment length polymorphism (AFLP) on resistant and susceptible maize and sorghum genotypes to identify differentially expressed genes, when challenged with *S. turcica*. This was followed by functional assessment of selected gene candidates by virus-induced gene silencing (VIGS) using a *Brome mosaic virus *vector. We found one *R*-gene cluster, containing six CC-NB-LRR encoding genes residing as three pairs in the sorghum genome, of importance for defense to *S. turcica*. Genome data further showed that the *St *genes are highly conserved within monocots.

## Results

### Identification of an up-regulated *R*-gene family in maize and sorghum in response to *S. turcica *inoculation

In order to identify important defense genes to *S. turcica*, cDNA-AFLP analysis was carried out on susceptible (S) and resistant (R) sorghum and maize genotypes following fungal infection. In our case, the Ugandan sorghum genotypes GA06/18 (R) and Sila (S) and the maize A619Ht1 (R) and A619 (S) lines were used. The sorghum material had earlier been evaluated on various agronomical traits including important fungal diseases. Apart from *S. turcica *responses, GA06/18 was found to be susceptible to *Cercospora sorghi*, and *Colletotrichum sublineolum*, whereas Sila was susceptible to *C. sorghi *and resistant to *C. sublineolum*.

In total, approximately 3000 transcript-derived fragments were monitored ranging from 50 to 600 bp in size using different primer combinations (Additional file 1). Unique, up- or down-regulated transcripts in the resistant genotypes compared to the susceptible, sampled at 24, 48 and 72 hours post inoculation (hpi) were excised, amplified, sequenced and analyzed for putative function. The final transcript-set comprised of 68 sorghum and 82 maize gene candidates. Among these genes, 11 and 13, respectively, were putative stress-related according to closest genes identified in other organisms using BLASTP.

**Figure 1:**
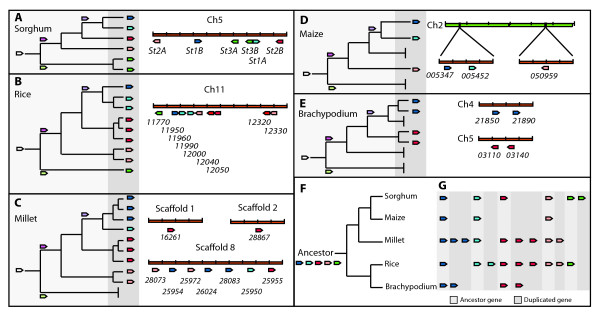
**Evolution of *St R*-gene cluster in monocots species**. Chromosome location, duplication and ancestry of the *St *gene cluster in A. sorghum, B. millet, C. rice, D. maize, and E. Brachypodium. Events preceding, (light grey) and post speciation (dark grey) are shown. F. Proposed ancestral *R*-gene cluster composition using an ancestral tree of grass species adapted from Bowman *et al*. [55], and phylogenetic analysis of homologous genes in each species. Genes colored in relation to *St *genes as follows; *St1 *blue, *St2 *red, and *St3 *green. Gene information is listed in Additional file 2.

**Figure 2:**
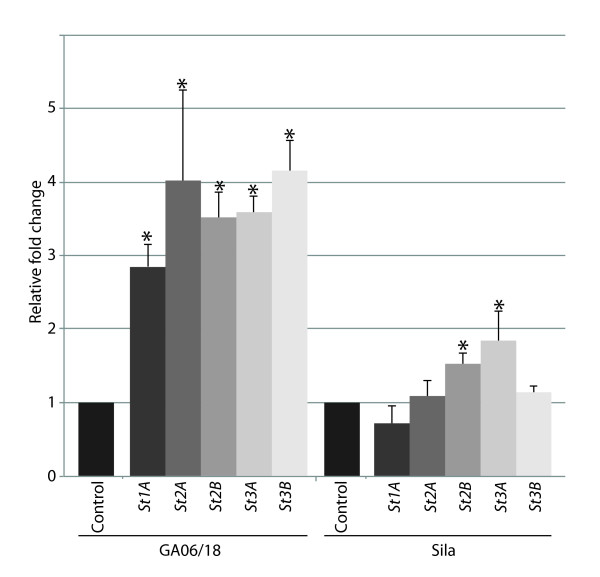
**Relative qPCR values of *St1A, St2A*, *St2B*, *St3A *and *St3B *transcripts in sorghum GA06/18 and Sila plants inoculated with *S. turcica*, 24 hpi**. Water treatment was used on respective genotypes as a control. Error bars indicate standard deviation between three biological replications. * Indicates a significant increase (*P *< 0.05) compared to control levels. Primer used listed in Additional file 2.

One CC-NB-LRR encoding putative *R*-gene (*GRMZM2G005347*), a member of a homologous gene pair with *GRMZM2G005452 *in the same locus on chromosome 2, and uniquely expressed in the resistant maize genotype, was further studied (Figure 1D). Genome analysis revealed presence of 6 homologous genes in sorghum (Figure 1A). These six genes were given the prefix *St *referring to *S. turcica *and designated *St1A *(*Sb05g008280*), *St1B *(*Sb05g008140*), *St2A *(*Sb05g008350*), *St2B *(*Sb05g008030*), *St3A *(*Sb05g008250*), and *St3B *(*Sb05g008270*). Quantitative real-time PCR confirmed furthermore that five (*St1A*, *St2A*, *St2B*, *St3A *and *St3B*) of the six *St *genes showed high relative transcript levels when the sorghum resistant GA06/18 plants were challenged with *S. turcica *(Figure 2). One gene, *St1B*, was expressed to a much lower extent compared to the other *St *genes, outside the limit of detection. In Sila, only *St2B *and *St3A *showed a significant increase (*P *< 0.005) in expressions when challenged with *S. turcica *(Figure 2).

### The *St *genes are conserved among grasses

The six *St *genes in sorghum form three gene pairs in a cluster on chromosome 5 and share a common ancestor (Figure 1; Additional file 2; Additional file 3). *St *gene orthologs were also found in clusters when searching the rice, maize, foxtail millet and Brachypodium genome databases. The *St *gene encoded proteins from the other grass species, grouped with the sorghum St proteins with high edge support (100) (Additional file 2). The rice genome contains orthologs of sorghum *St1A*, *St1B*, *St2A*, *St2B *and an *St3 *gene (Figure 1A, B). This indicates that the ancestor of rice and sorghum likely had a copy of these genes. Sorghum *St3A *and *St3B *are likely a result of a more recent genome duplication event after the split between the rice and sorghum species (Figure 1G). The rice genome also contains multiple copies of *St1A, St2A *and *St2B *orthologs, likely produced from gene duplications after the species split from sorghum. Likewise, the *Setaria italica *(foxtail millet) genome contains orthologs of *St1A*, *St1B*, *St2A *and *St2B*, with seven of the nine genes found in a cluster within the same scaffold, as complete chromosome annotation have yet to be determined (Figure 1C). An *St3 *homolog was not found in millet. In addition to the maize gene pair identified in our cDNA-AFLP analysis, BLASTP and BLASTN searches revealed a third single gene homolog, *GRMZM2G050959, St2A *on maize chromosome 2 (Figure 1D). The model grass Brachypodium genome, on the other hand, has a gene pair orthologous to *St1B *on chromosome 4, and one to *St2B *on chromosome 5, but lacks all other gene homologs (Figure 1E). The *St *gene cluster is maintained between sorghum, rice and possibly millet genomes but is smaller in maize and Brachypodium with *St *genes located across or on different chromosomes.

**Figure 3:**
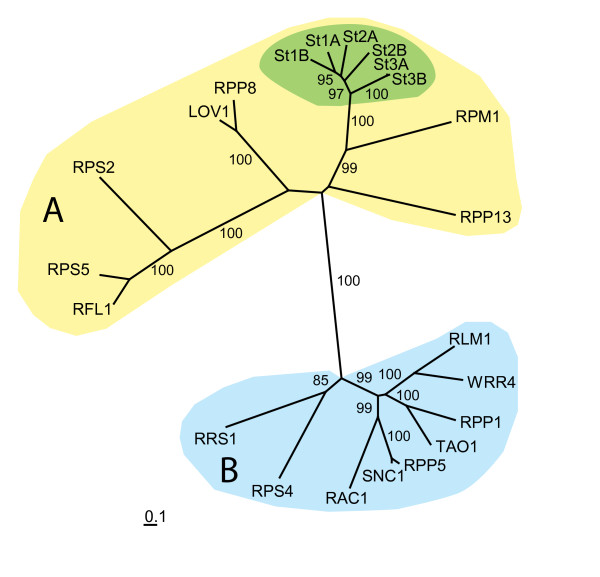
**Un-rooted maximum-likelihood phylogram inferred from nucleotide binding (NB) and leucine rich repeat (LRR) domains, of six resistance proteins to *S. turcica *(St) in sorghum, compared with Arabidopsis NB-LRR resistance proteins with known function**. LR-ELW values above 75% are shown. A. NB-LRR resistance proteins with a coiled-coil (CC) domain at the N-terminal end. B. NB-LRR resistance proteins with a Toll/Interleukin-1 receptor (TIR) at the N-terminal end. Units indicate substitutions/site. R-proteins used are listed in Additional file 4.

Sequence homology was also found between sorghum St proteins and Arabidopsis CC-NB-LRR encoding genes (Figure 3; Additional file 4). All six St proteins formed a cluster together with the CC rather than TIR domain containing R proteins from *Arabidopsis *indicating a closer evolutionary relationship as expected. The nearest related *Arabidopsis *gene is *RPM1*, a gene mediating resistance to *Pseudomonas syringae *isolates expressing the *avrRpml *or *avrB *genes [27].

### Adapting the VIGS system on sorghum

Genetic transformation of sorghum and maize is possible but laborious and requires other genotypes than those used in this study to be successful [28,29]. Hence, our candidate genes were further studied using virus induced gene silencing (VIGS) using the *Brome mosaic virus *(BMV) system, previously used to silence genes in monocots [30]. VIGS was followed by fungal inoculation to assess the potential defense function of the *St *genes. In our hands, the VIGS procedure was not successful when applied to the A619Ht1 maize genotype. Because the *St *genes were up-regulated upon fungal inoculation with *S. turcica *in our sorghum GA06/18 genotype (Figure 2), we continued the studies on our sorghum materials.

**Figure 4:**
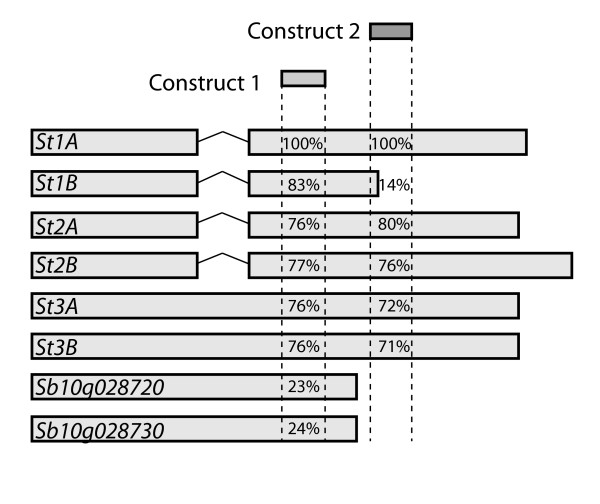
**Schematic alignment of VIGS constructs and *St *genes and their closest off-target genes**. Two sections of *St1A *were PCR amplified and ligated into VIGS plasmid pF13m. The identity of the constructs to each of the *St *genes is shown. E values for construct 1 and 2 against *St *and closest off target genes; *St1A *2e-128, 2e-128; *St 1B *4e-68, NA; *St2A *2e-34 5e-48; *St2B *1e-43 6e-60; *St3A *2e-33 8e-27; *St3B *3e-32 4e-24; *Sb10g028730 *3e-07, NA; *Sb10g028720 *3e-06, NA. NA indicates no significant similarity.

Two VIGS constructs (1 and 2) with high identity to the 6 *St *genes in sorghum were designed (Figure 4) including examination for their off-target gene silencing capacity. The highest non-*St *sorghum gene similarity belongs to a related *R*-gene pair, *Sb10g028720 *and *Sb10g028730*, located in a different subgroup upon phylogenetic analysis (Additional file 2), and used as a control for off-target gene silencing. The selected sequences were amplified and ligated into the third plasmid (pF13m) in the BMV system, and used to infect the sorghum plants.

**Figure 5:**
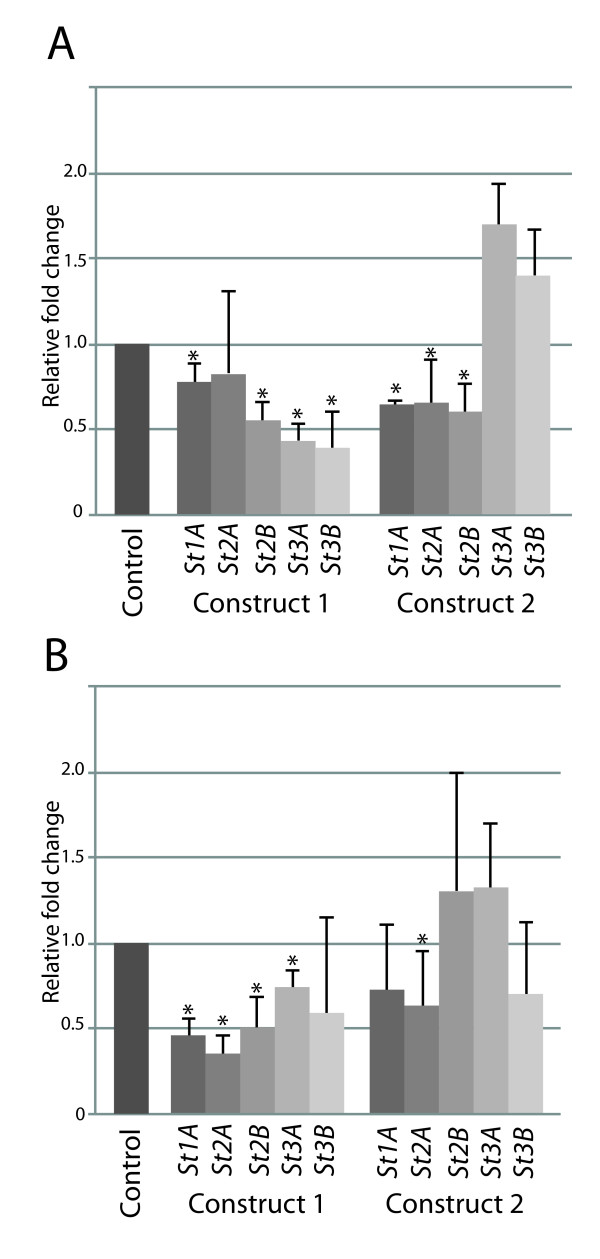
**Relative qPCR values of *St *gene transcripts when inoculated with VIGS constructs 1 and 2 compared to empty vector control**. Both GA06/18 (A) and Sila (B) genotypes showed down-regulation of five of the six *St *genes when inoculated with either one, or both of the constructs. Error bars indicate standard deviation between three biological replications. * Indicates a significant decrease (*P *< 0.05) compared to control levels.

The VIGS procedure was first optimized. Sorghum seeds were surface sterilized before sowing to minimize additional stress by other microorganisms. mRNA was produced by in vitro transcription, added to inoculation buffer and rubbed directly onto the second leaf of three week old sorghum plants. No intermediate step involving barley as virus host was used. The virus spreads systemically throughout the plant with silencing greatest in the second and third leaves above the inoculation site and complete silencing rarely achieved [30]. Seven days post infection (dpi), light green colored streaks were visible on the third leaf, indicating viral symptoms and successful infection by the virus. In order to confirm onset of silencing quantitative real time-PCR was carried out on leaf samples from the VIGS treated plants (Figure 5). There was a significant decrease in the relative transcript levels in relation to control plants inoculated with empty plasmid suggesting a clear down-regulation of five of the six targeted genes, particularly by construct 1, in both sorghum genotypes. Relative transcript levels of *Sb10g028720 *and *Sb10g028730 *were not influenced in VIGS treatments indicating no off-target silencing.

### Silencing of *St *genes increases *S. turcica *infection in the resistant and susceptible sorghum genotypes

**Figure 6:**
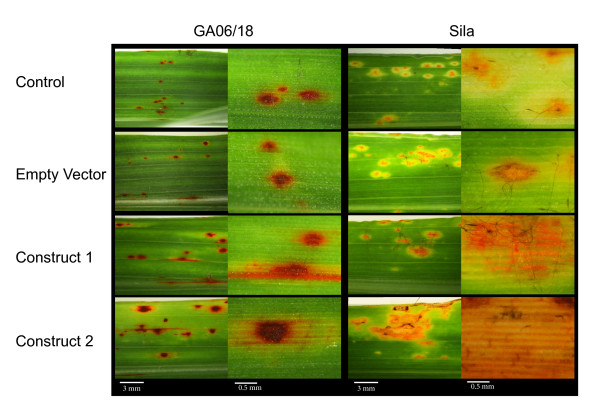
**Leaf phenotypes of resistant GA06/18 and susceptible Sila plants, 7 dpi with *S. turcica*, pretreated either with water, empty vector or VIGS construct 1 and 2**.

Fungal colonization and growth on plants inoculated with the different VIGS constructs compared with control material was carefully monitored. The different phenotypic observations are summarized in Figure 6; and Additional file 5. Fungal growth was further assessed by detaching infected leaves and placing them in a petri dish containing moist filter paper followed by incubation in the dark at 25°C for two days, as described by Levy [31]. The development of conidiophores protruding through leaf lesions followed by rapid asexual spore development indicated fungal colonization of the leaf material, and a susceptible phenotype.

**Figure 7:**
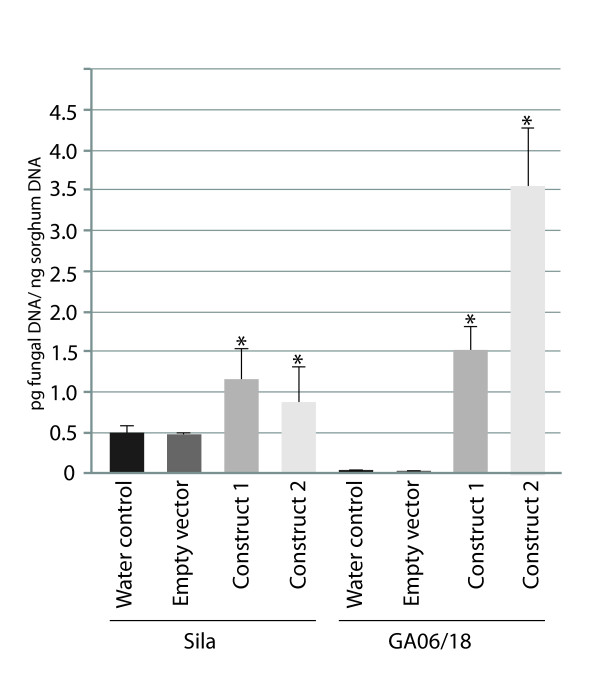
**Real-time qPCR assessment of *S. turcica *DNA on resistant GA06/18 and susceptible Sila leaf samples, 7 dpi with *S. turcica*, pretreated either with water, empty vector, or VIGS construct 1 and 2**. Error bars indicate standard deviation between three biological replications. * Indicates a significant increase (*P *< 0.05) in fungal DNA compared to control levels.

A hypersensitive response (small dark/red spots) occurred at 2 dpi on the resistant GA06/18 genotype upon fungal challenge while the plants treated with empty vector produced a somewhat delayed HR phenotype 3 dpi. When VIGS construct 1 was applied to GA06/18 plants prior to fungal inoculation, larger and more numerous lesions with chlorotic halos developed compared to the control plants. Disease lesions spread laterally along the leaf and fungal conidiophores and spores were produced under sporulating conditions. Similarly, when the effect of construct 2 was assayed, the disease lesions were seen 2 dpi and spread laterally to form large lesions that produced large numbers of fungal spores. The disease lesions were larger than those induced by construct 1, at 7 dpi. On the susceptible Sila plants clear disease symptoms, necrotic spots, and chlorotic halos around fungal appressoria were seen 2 dpi. Large numbers of asexual fungal spores were produced on conidiophores protruding from necrotic lesions. When Sila plants were inoculated with the empty VIGS vector, prior to fungal inoculation, similar disease symptoms occurred 2 dpi. In contrast, on Sila plants inoculated with our VIGS construct 1, slightly larger and more frequent lesions appeared compared to control plants. The disease symptoms were further amplified when construct 2 was used, resulting in larger necrotic lesions, and profuse fungal sporulation. In order to correlate these observed disease phenotypes with fungal growth, fungal DNA was quantified in the VIGS materials (Figure 7). *S. turcica *DNA increased to 1.5 ± 0.4 pg/ng sorghum DNA in GA06/18 leaves inoculated with VIGS construct 1, and to 3.6 ± 0.9 pg/ng sorghum DNA when using construct 2, from a near zero level in control plants (non-VIGS and empty vector). A significant (*P *< 0.005) increase in fungal DNA was also found in samples from Sila inoculated with construct 1 (1.2 ± 0.4 pg/ng sorghum DNA), and construct 2 (0.8 ± 0.9 pg/ng sorghum DNA), compared to control samples with approximately 0.5 pg/ng sorghum DNA.

Taken together, as expected the resistant GA06/18 genotype showed a compromised defense response when inoculated with VIGS construct 1 or 2 prior fungal inoculation. Interestingly, we observed enhanced disease phenotypes on the susceptible Sila genotype upon corresponding VIGS treatments.

## Discussion

Sorghum [*Sorghum bicolor *(L.) Moench] serves as a major food staple and fodder resource especially in arid and semi-arid regions of the world [32]. It is mainly a self-pollinating and diploid grass species (2n = 2x = 20), with a genome size of 1C = 730 Mbp, which is about 25% the size of the maize genome [4,5]. In the sorghum genome, 211 NB-LRR encoding *R*-genes are present, which is approximately half the number found in rice and slightly more compared to Arabidopsis [4]. The number of NB-LRR encoding genes in the small genome of the wild grass Brachypodium is estimated to 178 [6]. But in the much larger maize genome, 95 NB-LRR encoding genes have up to now been identified [33]. However, depending on search programs and threshold settings, slightly different *R*-gene numbers in each grass species are published.

It is postulated that the high numbers of *R*-genes in plant genomes and their large sequence diversity are essential evolutionary factors in the surveillance machinery to resist pathogen attacks. Resistance genes evolve through duplication, unequal crossing over, recombination and diversification leading to clusters of paralogous genes [10,34]. The proliferation of *R*-genes is also coupled with rapid turnover of gene copies, eventually leading to deletion or expansion and thus dynamic *R*-gene clusters [33]. Resistance gene clusters have also been found to be conserved between different species in Poaceae [35], although, such clusters are in the minority with 71.6% being specific to a species [33].

Whole genome duplications occurred when the grass subfamilies diverged from each other and genome data suggest further, that paleo-duplicated gene pairs in sorghum and rice remained extant in about 17% of the cases [36]. Recent duplications of chromosomal segments are particularly found on rice chromosomes 11 and 12, and corresponding regions on chromosome 5 and 8 in sorghum. Chromosome 5, in the sequenced BTx623 sorghum genotype, where the *St *genes are located showed the highest abundance (62) of *R*-genes [4]. Thirty-six of these NB-LRR encoding genes are affected by recent duplication events based on the bioinformatic analyses presented by Wang et al. [36], including *St3A *and *St3B*, which is in agreement with our results (Figure 1; Additional file 2). Interestingly, the rice genome contains orthologs of *St1A*, *St1B*, *St2A*, *St2B *and a single ortholog of the *St3 *genes, all in one single locus. This indicates that this gene cluster predates the species split of rice and sorghum. In the grass family, sorghum, maize and millets belong to the same sub-family (Panicoideae), whereas rice is located in Ehrhartoideae [37]. It is estimated that these two subfamilies diverged from a common ancestor 50-70 million years ago together with Pooideae, the subfamily to which Brachypodium, wheat, and barley belong.

In a genome-wide comparison of *Arabidopsis thaliana *and *A. lyrata*, the evolutionary pattern of the *R*-genes could be divided into two distinct groups, the positively selected (> 50%) with high sequence divergence between the two species, or the stably selected genes (< 30%) [38]. The remaining genes were only found in one genome and absent from the other. The *St *genes found in this work have experienced few sequence exchanges resulting in low divergence, and hence more resemble the description of stably selected genes, although the copy numbers vary between the five grass genomes compared (Figure 1). That NB-LRR encoded *R*-genes remain conserved between different grass species is presently believed to be a common phenomenon [33].

Sorghum plants, particularly genotypes with red seed color, accumulate a range of phenolic substances in response to pathogen attacks [39]. Large amounts of red-pigmented flavonoids induced at the site of infection were also seen in our materials, particularly in the resistant genotype. Whether flavonoids contribute to the defense response against *S. turcica *is not elucidated but a genetic link has been found in the sorghum - *C. sublineolum *interaction, produced via the presence of 3-deoxyanthocyanidins [40]. Reinforcement of plant cells via callose deposition upon pathogen attacks have been observed in many pathosystems. Enhanced callose deposition has also been reported as a resistance response to *S. turcica *in maize [41]. Despite extensive staining efforts, no callose accumulation was seen in either of our sorghum genotypes (data not shown).

Furthermore, our gene silencing work resulted in an enhanced susceptible response in Sila, our susceptible sorghum cultivar. This observation may suggest that by targeting the *St *genes in this genomic background, effects on downstream signaling masked in the resistant sorghum genotype are revealed, and could potentially constitute a fraction of the quantitative traits earlier found [41]. This hypothesis is speculative and remains to be included in future functional studies of the *St *genes. Future studies do also comprise a search for important effectors in the genome recently released from JGI http://www.jgi.doe.gov. In parallel, the sequence information from the *St *gene cluster is presently converted into molecular markers and used in germplasm assessments and breeding programs in East Africa, an important development to sustain sorghum and maize crop production in this part of the world.

## Conclusions

Our cDNA-AFLP analysis on susceptible and resistance maize and sorghum genotypes challenged by *S. turcica *resulted in identification of a CC-NB-LRR encoding gene in maize. This gene resides in two loci on maize chromosome 2. In sorghum, 6 *St *orthologous genes are present in a cluster of three pairs, on chromosome 5. Upon gene-silencing of the sorghum *St *genes, the resistance was clearly compromised, an observation that was supported by real-time PCR analysis and fungal DNA quantification. Database searches and phylogenetic analysis suggest that the *St *genes have a common ancestor present before the subfamily split, 50-70 million years ago, and the genes are highly conserved in sorghum, rice, foxtail millet, maize and Brachypodium.

## Methods

### Plant and fungal materials

Resistant (R) and susceptible (S) *Sorghum bicolor *genotypes from Uganda, GA06/18 (R) and Sila (S), and maize lines A619Ht1 (R) and A619 (S) provided by USDA ARS, were used in the study. The plants were grown in a growth chamber (Percival) using a 12/12 h photoperiod at 22°C. A single spore isolate from *S. turcica *infected sorghum (*Ig1*), or infected maize (*Mb1*), collected from Iganga and Mbale, Uganda, was used for all sorghum and maize analysis, respectively. The fungal DNA was extracted using a modified CTAB method [42]. DNA was analyzed by using *S. turcica *specific ITS1 and ITS2 primers (F -GCAACAGTGCTCTGCTGAAA and R-ATAAGACGGCCAACACCAAG). PCR was carried out using the following conditions: 10 ng of template DNA was added to a 24 μl mix consisting of H_2_O, 2.5 mM MgCl_2_, 2.5 μl *Taq *buffer (Fermentas, Helsingborg, Sweden) 0.2 mM of each dNTP, 0.25 μM of forward and reverse primers and 1 U of *Taq *polymerase (Fermentas) with: 3 min at 94°C, 35 cycles of (1 min at 94°C, 1 min at 60°C, and 1.5 min at 72°C), and final extension at 72°C for 10 min. The PCR products were separated on 1% agarose gels to confirm fragment size, (344 bp) followed by sequencing (Macrogen Inc., Seoul, Korea).

### Fungal inoculation of plant material

Three-week old seedlings were inoculated on the third leaf whorl with 25 μl conidia suspension (5 × 10^5 ^conidia/ml) as described by Carson [43]. Inoculated leaves from three to four plants were pooled and harvested at 24, 48, and 78 hours post inoculation (hpi) for cDNA-AFLP analysis. Water treated control samples were harvested at the same time-points.

### RNA extraction and cDNA-AFLP analysis

Total RNA was isolated from the leaf samples using the BioRad RNA isolation kit (BioRad, California, USA) followed by mRNA preparation with the mRNA capture kit (Roche, California, USA). cDNA was synthesized with Oligo-dT primer and RevertAid™ H Minus M-MuLV Reverse Transcriptase (Fermentas). Second strand was synthesized using *E. coli *DNA Polymerase I (Fermentas). The double stranded cDNA was digested with *Bst*Y1 and *Mse*1 (Fermentas) and ligated to respective adaptors, pre-amplified and later selectively amplified using the *Bst*YI +N (^33^P labeled) and *Mse*I +N primers. Pre-amplification was carried out with the adapter-ligated cDNA, Taq DNA Polymerase (Fermentas) and the non-selective primers specific to the *Bst*YI and *Mse*I adapters using 25 cycles of 94°C for 30 s; 56°C for 1 min and 72°C for 1 min. The pre-amplified reaction mixture was diluted 600-fold and 5 μl was used for final selective amplification with 24 primer combinations, carried out with *Bst*YI +N (^33^P labeled) primers (Additional file 1) and touchdown amplification [44]. The selective amplification products were resolved on 6% polyacrimide gel run at 100 W until 4300 Vh was reached. Gels were dried and exposed to Kodak Biomax film (Amersham Pharmacia, California, USA) for 5-7 days.

### Isolation and sequencing of transcripts

Approximately 150 transcripts (unique, up and down-regulated) from the resistant genotypes in relation to the susceptible genotypes, were excised from the dried PAGE gels, eluted in H_2_O and PCR amplified using the non-selective primers under the same conditions as earlier described in the pre-amplification step. The products were cloned into the pJET 1.2 blunt vector (CloneJET™ PC, Fermentas) and sequenced. The sequences were analyzed using the BLASTN and BLASTX programs [45] and compared with sequences deposited in NCBI, GRAMENE and PHYTOZOME databases. Identified fungal sequences were excluded.

### Virus induced gene silencing (VIGS) in sorghum

The VIGS system used is based on the monocot-infecting *Brome mosaic virus *(BMV) as previously described [30] but pre-inoculation on barley was excluded. The BMV VIGS vector consists of three plasmids harboring BMV RNA1 (p1-1), RNA2 (p2-2) and RNA3 (pF13m, formally pF3-5/13_A/G_), respectively. To generate VIGS constructs, PCR fragments ranging from 246 to 253 bp in size were amplified from the sorghum candidate gene using genomic DNA of the resistant GA06/18 genotype and gene-specific primers harboring *Nco*I and *Avr*II restriction sites using the Primer 3 version 0.4.0 http://frodo.wi.mit.edu/primer3/ software (Additional file 6). Prior to PCR amplification, off-target gene searches were undertaken to design optimal VIGS constructs (Figure 4). After restriction, each fragment was cloned into the corresponding site of the pF13m plasmid. The identity of the inserts was verified by sequencing. P1-1, p2-2 and the pF13m containing different constructs were digested with *Spe*I, *Psh*AI and *Psh*AI, respectively. Infectious RNA transcripts were synthesized from linearized plasmids through *in vitro *transcription using T3 Polymerase (Fermentas), according to manufacturer instructions. 1 μl of the reaction product was run on a 1.5% agarose gel to confirm presence of a transcript.

Plant inoculation procedures were performed as described [30] with slight modifications. A 10 μl aliquot of the transcription mix from each of the plasmids p1-1, p2-2 and pF13m-insert was combined with 30 μl FES inoculation buffer and used directly to rub inoculate the second and third leaves of 3-week-old sorghum and maize plants. As a control, plants were inoculated in the same way with water or combined transcripts from p1-1, p2-2 and empty pF13m. Maize and sorghum plants were challenged with *S. turcica *as earlier described one week after viral inoculation (when faint chlorosis and vein clearing started to appear) to assess the effect of the different constructs. Plants were randomized and coded to reduce potential bias in the scoring of fungal colonization and growth.

### Quantitative real-time PCR

Prior to fungal inoculation of the VIGS treated sorghum plants, approximated 3 cm of the second leaf above the VIGS inoculated leaf was collected from 3 independent plants in triplicates for each condition and used for RNA extraction as previously described. First strand-cDNA was synthesized from 1 μg of total RNA, with Oligo-dT primer and RevertAid™ H Minus M-MuLV Reverse Transcriptase (Fermentas) according to the manufacturer's instructions. Real-time PCR was carried out using the first strand cDNA in an iQ5 cycler (Bio-Rad). Maxima Sybr Green/Fluorescein qPCR Master Mix (Fermentas) was used for PCR amplification in a 20 μl total reaction volume consisting of 10 μl of SYBR Green qPCR Master Mix, 0.3 μM forward and reverse primers and 5 ng of cDNA template. All PCRs were performed in triplicate under the following amplification conditions; 10 min at 95°C followed by 40 cycles of 95°C, for 15 s, 60°C for 30 s, and 72°C for 30 s, followed 1 min at 95°C, and melt curve analysis. Primers sequences for *St *genes were designed using the Primer 3 version 0.4.0 http://frodo.wi.mit.edu/primer3/ software (Additional file 7). The sorghum elongation factor 1-alpha (*Sb02g036420*) and Actin (*Sb01g010030*) were used as reference genes and relative transcript values were calculated. All calculations and statistical analyses were performed as described in the ABI PRISM 7700 Sequence Detection System User Bulletin #2 (Applied Biosystems, USA) slightly modified by Vetukuri et al. [46]. Quantification of *S. turcica *DNA on VIGS material 7 days post fungal inoculation was carried out as earlier described [41]. Approximately 3 cm of leaf material from three plants was pooled and DNA extracted. Three biological samples per treatment were analyzed. Statistical significance was calculated using Student's t-test.

### Genome analysis

The amino acid sequences of *St1A *(*Sb05g008280*), *St1B *(*Sb05g008140*), *St2A *(*Sb05g008350*), *St2B *(*Sb05g008030*), *St3A *(*Sb05g008250*) and *St3B *(*Sb05g008270*) were aligned to sorghum, maize, millet, rice, Brachypodium and Arabidopsis genome databases using BLASTN and BLASTP (PHYTOZOME). Predicted domains were identified using coiled-coil prediction [47]), LRRfinder [48] and CD-Search [49]. *St*-like gene loci were identified using Genomic Viewer (PHYTOZOME). Phylogenetic analysis was conducted using Treefinder and maximum likelihood and 10, 000 replicates [50]. The JTT+G model [51] was found to best fit the data using ProtTest v2.4 [52]. Confidences were calculated using local rearrangement of expected likelihood weights (LR-ELW) [53]. Phylograms were drawn using Treeview 1.6.6 [54].

## Authors' contributions

TM carried out the qPCR, genomic analysis, created figures, and ran the cDNA-AFLP and VIGS analyses together with MB. IF supported on the VIGS analysis. CD and PO conceived the study and participated in writing together with all authors. All authors read and approved the final manuscript.

## Supplementary Material

Additional file 1 **PCR primer combinations used in cDNA-AFLP analysis**. Click here for file

Additional file 2 **Maximum likelihood phylogenetic tree using the model JTT+G based on amino acid sequence from the coiled coil (CC), nucleotide binding (NB) and leucine rich repeat (LRR) domains of St proteins in sorghum, and closely related R proteins**. Names refer to PHYTOSOME gene identifier. *Physcomitrella patens R*-protein Pp1s1_327V6, was used as an out-group. LR-ELW edge support values are shown [53]. Substitutions per site are indicated. Click here for file

Additional file 3 **Information on genes used in Figure **1** and their putative function**. Data retrieved from the PHYTOSOME database. GenBank accession numbers are stated where present. Click here for file

Additional file 4 **Information on Arabidopsis genes used in **Figure 3. **Data retrieved from the TAIR database**. Click here for file

Additional file 5 **Disease phenotypes on sorghum leaves, monitored 1-12 days post inoculation (dpi) with *S. turcica *on the resistant wild type GA06/18 and the susceptible Sila cultivar**. The plants were treated with either water, empty BMV vector, construct 1 or construct 2, prior to fungal inoculation. The data is compiled from 25-30 plants per BMV construct and controls. The experiment was repeated 2 times. Click here for file

Additional file 6 **Gene specific primers for VIGS constructs. Restriction sites are in bold**. Click here for file

Additional file 7 **List of primers used in real time PCR analysis**. Click here for file

## References

[B1] Jones JDG Dangl J The plant immune system Nature 2006 444 7117 323 329 10.1038/nature05286 17108957

[B2] Meyers BC Kozik A Griego A Kuang H Michelmore RW Genome-wide analysis of NB-LRR-encoding genes in Arabidopsis Plant Cell 2003 15 4 809 834 10.1105/tpc.009308 12671079 PMC152331

[B3] Liu J Liu X Dai L Wang G Recent progress in elucidating the structure, function and evolution of disease resistance genes in plants Journal of Genetics & Genomics 2007 34 9 765 776 10.1016/S1673-8527(07)60087-3 17884686

[B4] Paterson AH Bowers JE Bruggmann R The *Sorghum bicolor *genome and the diversification of grasses Nature 2009 457 7229 551 556 10.1038/nature07723 19189423

[B5] Schnable PS Ware D Fulton RS The B73 maize genome: complexity, diversity and dynamics Science 2009 326 5956 1112 1115 10.1126/science.1178534 19965430

[B6] The International Brachypodium Initiative Genome sequence and analysis of the model grass *Brachypodium distachyon* Nature 2010 463 7282 763 768 10.1038/nature08747 20148030

[B7] Collier SM Moffett P NB-LRRs work a "bait and switch" on pathogens Trends in Plant Science 2009 14 10 521 529 10.1016/j.tplants.2009.08.001 19720556

[B8] Smith SM Pryor AJ Hurlbert SH Allelic and haplotypic diversity at the *rp1 *rust resistance locus of maize Genetics 2004 167 4 1939 1949 10.1534/genetics.104.029371 15342531 PMC1471013

[B9] Kuang H Woo S-S Meyers BC Nevo E Michelmore RW Multiple genetic processes result in heterogeneous rates of evolution within the major cluster disease resistance genes in lettuce Plant Cell 2004 16 11 2870 2894 10.1105/tpc.104.025502 15494555 PMC527186

[B10] McDowell JM Simon SA Recent insights into *R *gene evolution Molecular Plant Patholology 2006 7 5 437 448 10.1111/j.1364-3703.2006.00342.x 20507459

[B11] Dixon MS Jones DA Keddie JS Thomas CM Harrison K Jones JDG The tomato *Cf-2 *disease resistance locus comprises two functional genes encoding leucine-rich repeat proteins Cell 1996 84 3 451 459 10.1016/S0092-8674(00)81290-8 8608599

[B12] Eitas TK Dangl JL NB-LRR proteins: pairs, pieces, perception, partners and pathways Current Opinion in Plant Biology 2010 13 4 472 477 10.1016/j.pbi.2010.04.007 20483655 PMC2910844

[B13] Hamid A Aragaki M Inheritance of pathogenicity in *Setosphaeria turcica* Phytopathology 1975 65 3 280 283 10.1094/Phyto-65-280

[B14] Chiang M van Dyke C Leonard K Evaluation of endemic fungi for potential biological control of Johansongrass (*Sorghum halepense*): Screening and host range tests Plant Disease 1989 73 6 459 464 10.1094/PD-73-0459

[B15] Perkins JM Pedersen WL Disease development and yield losses associated with northern leaf blight on corn Plant Disease 1987 71 10 940 943 10.1094/PD-71-0940

[B16] Carson ML van Dyke C Effect of light and temperature on expression of partial resistance of maize to *Exserohilum turcicum* Plant Disease 1994 78 4 408 411

[B17] Pratt R Gordon S Breeding for resistance to maize foliar pathogens Plant Breeding Reviews 2006 27 119 173

[B18] Welz G Geiger H Genes for resistance to northern corn leaf blight in diverse maize populations Plant Breeding 2000 119 1 1 14 10.1046/j.1439-0523.2000.00462.x

[B19] Mangelsdorf PC Corn: its origin, evolution and improvement 1974 Belknap Press, Cambridge, Mass. USA

[B20] Kimber CT Smith CW, Frederiksen RA Origins of domesticated sorghum and its early diffusion into India and China Sorghum: origin, history, technology and production 2000 John Wiley & Sons, New York, USA 3 98

[B21] Ngugi HK King SB Holt J Julian AM Simultaneous temporal progress of sorghum anthracnose and leaf blight in crop mixtures with disparate patterns Phytopathology 2001 91 8 720 729 10.1094/PHYTO.2001.91.8.720 18944028

[B22] Adipala E Lipps EP Madden LV Occurrence of *Exserohilum turcicum *on maize in Uganda Plant Disease 1993 77 1 202 205

[B23] Ebiyau J Oryokot OE Sorghum (*Sorghum bicolor *(L.) Moench. Agriculture in Uganda 2001 II Crops: National Agricultural Research Organisation Fountain Publ

[B24] Muiru WM Histological studies and characterization of races of *Exserohilum turcicum *the causal maize agent of northern leaf blight of maize in Kenya PhD thesis 2008 University of Nairobi, Kenya

[B25] Ramathani I Characterisation of turcicum leaf blight epidemics and pathogen populations in the *Exserohilum turcicum *- Sorghum pathosystem in Uganda MSc thesis 2009 Makerere Univ. Kampala, Uganda

[B26] Ramathani I Biruma M Martin T Dixelius C Okori P Disease severity, incidence and races of *Setosphaeria turcica *on sorghum in Uganda European Journal of Plant Pathology 2011 131 3 383 392 10.1007/s10658-011-9815-1

[B27] Grant MR Godiardt L Straube S Ashfield T Lewald J Satler A Ines RW Dangl JL Structure of the *RPM1 *gene enabling dual specificity disease resistance Science 1995 269 5225 843 846 10.1126/science.7638602 7638602

[B28] Opabode J Agrobacterium-mediated transformation of plants: emerging factors that influence efficiency Biotechnology and Molecular Biology Reviews 2006 1 1 12 20

[B29] Gurel S Gurel E Kaur R Wong J Meng L Tan HQ Lemaux PG Efficient, reproducible *Agrobacterium*-mediated transformation of sorghum using heat treatment of immature embryos Plant Cell Reports 2009 28 3 429 444 10.1007/s00299-008-0655-1 19115059

[B30] Ding XS Schneider WL Chaluvadi SR Mian MAR Nelson RS Characterization of a *Brome mosaic virus *strain and its use as vector for gene silencing in monocotyledonous hosts Molecular Plant-Microbe Interactions 2006 19 11 1229 1239 10.1094/MPMI-19-1229 17073305

[B31] Levy Y Variation of fitness among field isolates of *Exserohilum turcicum *in Israel Plant Disease 1991 75 2 163 166 10.1094/PD-75-0163

[B32] Doggett H Sorghum 1988 2 John Wiley, New York, US

[B33] Li J Ding J Zhang Y Wang J Chen J-Q Tian D Yang S Unique evolutionary pattern of numbers of gramineous NBS-LRR genes Molelucar Genetics & Genomics 2010 283 5 427 438 10.1007/s00438-010-0527-6 20217430

[B34] Meyers BC Kaushik S Nandety RS Evolving disease resistance genes Current Opinion in Plant Biology 2005 8 2 129 134 10.1016/j.pbi.2005.01.002 15752991

[B35] Luo S Peng J Li K Wang M Kuang H Contrasting Evolutionary patterns of the *Rp1 *resistance gene family in different species of Poaceae Molecular Biology & Evolution 2011 28 1 313 325 10.1093/molbev/msq216 20713469

[B36] Wang X Wang H Paterson AH Seventy million years of concerted evolution of a homoeologous chromosome pair, in parallel, in major Poaceae lineages Plant Cell 2011 23 1 27 37 10.1105/tpc.110.080622 21266659 PMC3051248

[B37] Bolot S Abrouk M Masood-Quraishi U Stein N Messing J Feuillet C Salse J The inner circle of the cereal genomes Current Opinion in Plant Biology 2009 12 2 119 125 10.1016/j.pbi.2008.10.011 19095493

[B38] Chen Q Han Z Jiang H Tian D Yang S Strong positive selection drives rapid diversification of *R*-genes in *Arabidopsis *relatives Journal of Molecular Evolution 2010 70 2 137 148 10.1007/s00239-009-9316-4 20044783

[B39] Dicko MH Gruppen H Barro C Traore AS van Berkel WJH Voragn AGJ Impact of phenolic compounds and related enzymes in sorghum varieties for resistance and susceptibility to biotic and abiotc stresses Journal of Chemical Ecology 2005 31 11 2671 2687 10.1007/s10886-005-7619-5 16273434

[B40] Ibraheem F Gaffoor I Chopra S Phytoalexin-dependent resistance to anthracnose leaf blight requires a functional yellow *seed1 *in *Sorghum bicolor* Genetics 2010 184 4 915 926 10.1534/genetics.109.111831 20083611 PMC2865927

[B41] Chung C-L Longfellow JM Walsh EK Kerdieh Z van Esbroeck G Balint-Kurti P Nelson RJ Resistance loci affecting distinct stages of fungal pathogenesis: use of introgression lines for QTL mapping and characterization in the maize-*Setosphaeria turcica *pathosystem BMC Plant Biology 2010 10 103 10.1186/1471-2229-10-103 20529319 PMC3017769

[B42] Okori P Rubaihayo PR Ekwamu A Fahleson J Dixelius C Genetic characterization of *Cercospora sorghi *from cultivated and wild sorghum and its relationship to other *Cercospora *fungi Phytopathology 2004 94 7 743 750 10.1094/PHYTO.2004.94.7.743 18943907

[B43] Calsson ML Inheritance of latent period length in maize infected with *Exserohilum turcicum* Plant Disease 1995 79 6 581 585 10.1094/PD-79-0581

[B44] Vos P Hogers R Bleeker M Reijans M Vandelee T Hornes M Frijters A Pot J Peleman J Kuiper M Zabeau M AFLP a new technique for DNA fingerprinting Nucleic Acids Research 1995 23 21 4407 4414 10.1093/nar/23.21.4407 7501463 PMC307397

[B45] Altschul SF Gish W Miller W Myers EW Lipman DJ Basic local alignment search tool Journal of Molecular Biology 1990 215 3 403 410 2231712 10.1016/S0022-2836(05)80360-2

[B46] Vetukuri RR Avrova AO Grenville-Briggs LJ van West P Söderbom F Savenkov EI Whisson SE Dixelius C Evidence for involvement of Dicer-like, Argonaute, and Histone Deacetylase proteins in gene silencing in *Phytophthora infestans* Molecular Plant Pathology 2011 12 8 772 785 10.1111/j.1364-3703.2011.00710.x 21726377 PMC6640358

[B47] Lupas A Van Dyke M Stock J Predicting coiled coils from protein sequences Science 1991 252 5010 1162 1164 2031185 10.1126/science.252.5009.1162

[B48] Offord V Coffey TJ Werling D LRRfinder: a web application for the identification of leucine-rich repeats and an integrative Toll-like receptor database Development & Comparative Immunology 2010 34 10 1035 1041 10.1016/j.dci.2010.05.004 20470819

[B49] Marchler-Bauer A Bryant SH CD-Search: protein domain annotations on the fly Nucleic Acids Research 2004 32 W327 331 10.1093/nar/gkh454 15215404 PMC441592

[B50] Jobb G von Haeseler A Strimmer K TREEFINDER: a powerful graphical analysis environment for molecular phylogenetics BMC Evolutionary Biology 2004 4 18 10.1186/1471-2148-4-18 15222900 PMC459214

[B51] Jones DT Taylor WR Thornton JM The rapid generation of mutation data matrices from protein sequences Computer Applications in the Biosciences 1992 8 3 275 282 1633570 10.1093/bioinformatics/8.3.275

[B52] Abascal F Zardoya R Posada D ProtTest: selection of best-fit models of protein evolution Bioinformatics 2005 21 9 2104 2105 10.1093/bioinformatics/bti263 15647292

[B53] Strimmer K Rambaut A Inferring confidence sets of possibly misspecified gene trees Proceedings of the Royal Society B: Biological Sciences 2002 269 1487 137 142 10.1098/rspb.2001.1862 11798428 PMC1690879

[B54] Page RD TreeView: an application to display phylogenetic trees on personal computers Computer Applications in Biosciences 1996 12 4 357 358 10.1093/bioinformatics/12.4.357 8902363

[B55] Bowman J Floud S Sakakibara K Green genes - comparative genomics of the green branch of life Cell 2007 129 2 229 234 10.1016/j.cell.2007.04.004 17448980

