# Supplementary material for: A highly conserved NB-LRR encoding gene cluster effective against Setosphaeria turcica in sorghum
Source: BMC Plant Biol. 2011 Nov 3;11:151. doi: 10.1186/1471-2229-11-151 (PMC3262770; doi:10.1186/1471-2229-11-151)
Supplement: Additional file 1 — PCR primer combinations used in cDNA-AFLP analysis. [file 1471-2229-11-151-S1.DOC]

**Additional file 1:** Primer combinations used in cDNA–AFLP analysis.

| **Primer combination** | ***Bst*YI sequence** | ***Mse*I sequence** |
| --- | --- | --- |
| *Bst*YT3 + *Mse*3 | GACTCCGTAGTCATCTG | GATGAGTCCTGAGTAAG |
| *Bst*YT3 + *Mse*4 | GACTCCGTAGTCATCTG | GATGAGTCCTGAGTAAT |
| *Bst*YT42+ *Mse*13 | GACTGCGTAGTGATCTTC | GATGAGTCCTGAGTAAAG |
| *Bst*YT44 + *Mse*13 | GACTGCGTAGTGATCTTT | GATGAGTCCTGAGTAAAG |
| *Bst*YT43 + *Mse*13 | GACTGCGTAGTGATCTTG | GATGAGTCCTGAGTAAAG |
| *Bst*YT43 + *Mse*21 | GACTGCGTAGTGATCTTG | GATGAGTCCTGAGTAACA |
| *Bs*tYT43 + *Mse*4 | GACTGCGTAGTGATCTTG | GATGAGTCCTGAGTAAT |
| *Bst*YT44 + *Mse*4 | GACTGCGTAGTGATCTTT | GATGAGTCCTGAGTAAT |
| *Bst*YT13 + *Mse*21 | GACTGCGTAGTGATCTAG | GATGAGTCCTGAGTAACA |
