# Supplementary figures and images for: A highly conserved NB-LRR encoding gene cluster effective against Setosphaeria turcica in sorghum
Source: BMC Plant Biol. 2011 Nov 3;11:151. doi: 10.1186/1471-2229-11-151 (PMC3262770; doi:10.1186/1471-2229-11-151)

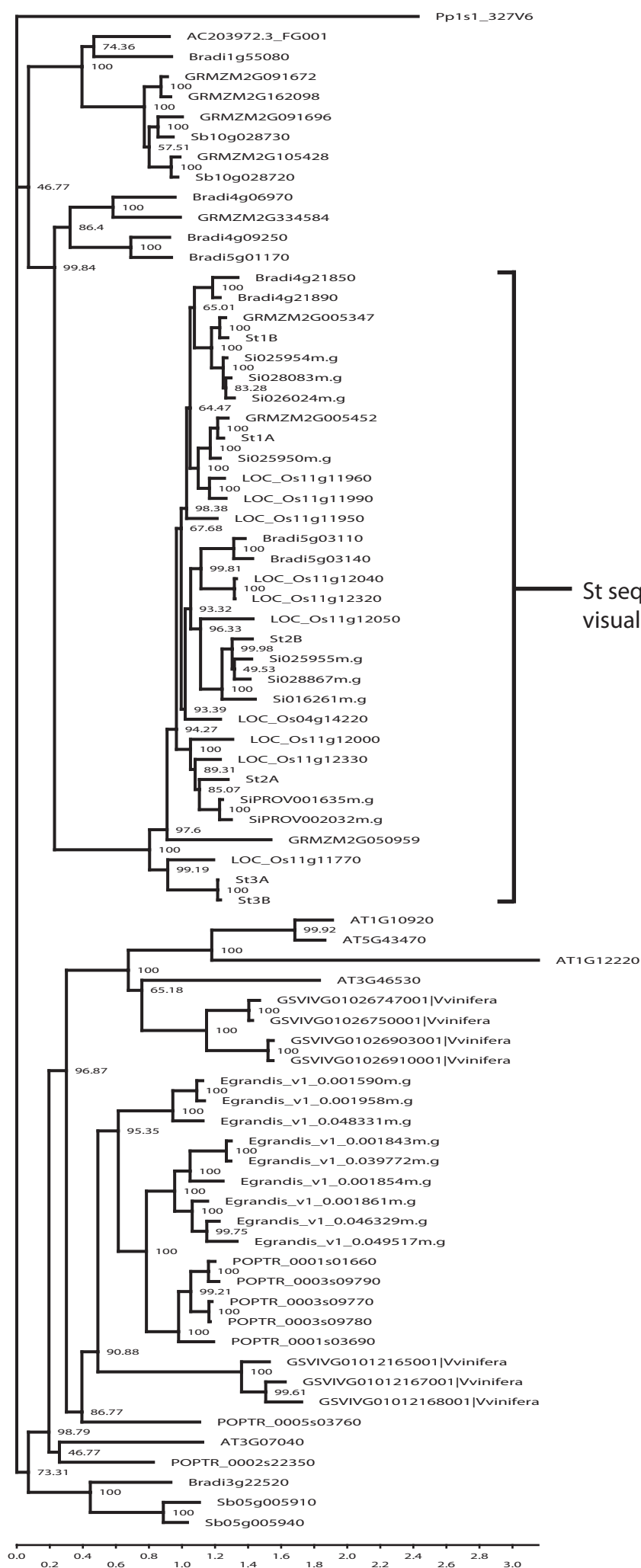

Supplement: Additional file 2 — Maximum likelihood phylogenetic tree using the model JTT+G based on amino acid sequence from the coiled coil (CC), nucleotide binding (NB) and leucine rich repeat (LRR) domains of St proteins in sorghum, and closely related R proteins. Names refer to PHYTOSOME gene identifier. Physcomitrella patens R-protein Pp1s1_327V6, was used as an out-group. LR-ELW edge support values are shown [53]. Substitutions per site are indicated. [file 1471-2229-11-151-S2.PDF]
