# Supplementary material for: A highly conserved NB-LRR encoding gene cluster effective against Setosphaeria turcica in sorghum
Source: BMC Plant Biol. 2011 Nov 3;11:151. doi: 10.1186/1471-2229-11-151 (PMC3262770; doi:10.1186/1471-2229-11-151)
Supplement: Additional file 3 — Information on genes used in Figure 1 and their putative function. Data retrieved from the PHYTOSOME database. GenBank accession numbers are stated where present. [file 1471-2229-11-151-S3.DOC]

**Additional file 6:** Genes used in in figure 1 and putative function.

|  | **Locus number** | **Gene name/ Predicted function** | **Chromosome** | **Accession^1^** |
| --- | --- | --- | --- | --- |
| *Sorghum bicolor* | |  |  |  |
|  | Sb05g008280 | *St1A* | 5 | XP_002449338.1 |
|  | Sb05g008140 | *St1B* | 5 | XP_002449331.1 |
|  | Sb05g008030 | *St2A* | 5 | XP_002450600.1 |
|  | Sb05g008350 | *St2B* | 5 | XP_002450621.1 |
|  | Sb05g008250 | *St3A* | 5 | XP_002450616.1 |
|  | Sb05g008270 | *St3B* | 5 | XP_002450618.1 |
| *Setaria italica^2^* | |  |  |  |
|  | Si016261m.g | Apoptotic ATPase |  |  |
|  | Si025950m.g | Apoptotic ATPase |  |  |
|  | Si025954m.g | Apoptotic ATPase |  |  |
|  | Si025955m.g | Apoptotic ATPase |  |  |
|  | Si025972m.g | Apoptotic ATPase |  |  |
|  | Si026024m.g | Apoptotic ATPase |  |  |
|  | Si028073m.g | Apoptotic ATPase |  |  |
|  | Si028083m.g | Apoptotic ATPase |  |  |
|  | Si028867m.g | Apoptotic ATPase |  |  |
| *Oryza sativa* | |  |  |  |
|  | LOC_Os11g11960 | NBS-LRR type disease resistance protein, putative, expressed | 11 | ABA92223.2 |
|  | LOC_Os11g11990 | NB-ARC domain containing protein, expressed | 11 | ABA92226.1 |
|  | LOC_Os11g11950 | NB-ARC domain containing protein, expressed | 11 | ABA92222.1 |
|  | LOC_Os11g12040 | NB-ARC domain containing protein, expressed | 11 | ABA92231.1 |
|  | LOC_Os11g12320 | Leucine Rich Repeat family protein | 11 | ABA92163.1 |
|  | LOC_Os11g12050 | NBS-LRR type disease resistance protein, putative, expressed | 11 | ABA92232.1 |
|  | LOC_Os11g12000 | NBS-LRR disease resistance protein, putative, expressed | 11 | ABA92227.1 |
|  |  |  |  |  |
|  | LOC_Os11g12330 | NB-ARC domain containing protein, expressed | 11 | ABA92164.1 |
|  | LOC_Os11g11770 | NB-ARC domain containing protein, expressed | 11 | ABA92204.1 |
| *Zea mays* | |  |  |  |
|  | GRMZM2G005347 | NBS-LRR type disease resistance protein | 2 |  |
|  | GRMZM2G005452 | NBS-LRR type disease resistance protein (MRPR1) | 2 | NM_001112339.1 |
|  | GRMZM2G050959 | NBS-LRR type disease resistance protein | 2 |  |
| *Brachypodium distachyon* | |  |  |  |
|  | Bradi4g21850 | LEUCINE-RICH REPEAT-CONTAINING PROTEIN | 4 |  |
|  | Bradi4g21890 | LEUCINE-RICH REPEAT-CONTAINING PROTEIN | 4 |  |
|  | Bradi5g03110 | LEUCINE-RICH REPEAT-CONTAINING PROTEIN | 5 |  |
|  | Bradi5g03140 | LEUCINE-RICH REPEAT-CONTAINING PROTEIN | 5 |  |

^1^NCBI Accession numbers

^2^Chromosome location undetermined
