# Supplementary material for: A highly conserved NB-LRR encoding gene cluster effective against Setosphaeria turcica in sorghum
Source: BMC Plant Biol. 2011 Nov 3;11:151. doi: 10.1186/1471-2229-11-151 (PMC3262770; doi:10.1186/1471-2229-11-151)
Supplement: Additional file 4 — Information on Arabidopsis genes used in Figure 3. Data retrieved from the TAIR database. [file 1471-2229-11-151-S4.DOC]

**Additional file 7:** *Arabidopsis thaliana* gene information used in figure 3.

| **Gene name** | **Abbreviation** | | | ***Accession** |
| --- | --- | --- | --- | --- |
| Locus orchestrating victorin effects 1 | | *LOV1* | ABW24145.1 | |
| Resistance to *Albugo candida* | | *RAC1* | AY522496.1 | |
| RPS5-like 1 | | *RFL1* | AAL65604.1 | |
| Resistance to *Leptosphaeria maculans* 1 | | *RLM1* | NP_176590.1 | |
| Resistance to *Pseudomonas syringae pv maculicola* 1 | | *RPM1* | NP_187360.1 | |
| Recognition of *Peronospora parasitica* 1 | | *RPP1* | NP_190034.2 | |
| Recognition of *Peronospora parasitica* 5 | | *RPP5* | AC074360.5 | |
| Recognition of *Peronospora parasitica* 8 | | *RPP8* | NP_199160.1 | |
| Recognition of *Peronospora parasitica* 13 | | *RPP13* | ABF00977.1 | |
| Resistant to *Pseudomonas syringae* 2 | | *RPS2* | NP_194339.1 | |
| Resistant to *Pseudomonas syringae* 4 | | *RPS4* | NP_199338.1 | |
| Resistant to *Pseudomonas syringae* 5 | | *RPS5* | NP_172686.1 | |
| Resistant to *Ralstonia solanacearum* 1 | | *RRS1* | NP_199339.1 | |
| Suppressor of NPR1-1, constitutive 1 | | *SNC1* | NP_193422.1 | |
| Target of AVRb operation 1 | | *TAO1* | NP_199264.1 | |
| White rust resistance 4 | | *WRR4* | NP_176043.1 | |

*NCBI Accession numbers
