# Supplementary material for: A highly conserved NB-LRR encoding gene cluster effective against Setosphaeria turcica in sorghum
Source: BMC Plant Biol. 2011 Nov 3;11:151. doi: 10.1186/1471-2229-11-151 (PMC3262770; doi:10.1186/1471-2229-11-151)
Supplement: Additional file 5 — Disease phenotypes on sorghum leaves, monitored 1-12 days post inoculation (dpi) with S. turcica on the resistant wild type GA06/18 and the susceptible Sila cultivar. The plants were treated with either water, empty BMV vector, construct 1 or construct 2, prior to fungal inoculation. The data is compiled from 25-30 plants per BMV construct and controls. The experiment was repeated 2 times. [file 1471-2229-11-151-S5.DOC]

**Additional file 3: Disease phenotypes on sorghum leaves, 7 dpi.**

|  | **VIGS construct** | **HR response** | **Infection symptoms** | | **Sporulation** |
| --- | --- | --- | --- | --- | --- |
| Sila (S)* | |  |  | |  |
|  | H_2_O control | No | Multiple necrotic lesions with chloritic halo, spreading lesions, 1-5 mm long | | Yes |
|  | Empty | No | Multiple necrotic lesions with chloritic halo, spreading lesions 1-5 mm long | | Yes |
|  | 1 | No | Multiple necrotic lesions with chloritic halo, spreading lesions 1-8 mm long | | Yes |
|  | 2 | No | Large necrotic lesions with chloritic halo, spreading lesions 1-12 mm long | | Profuse |
| GA06/18 (R) | |  | |  |  |
|  | H_2_O control | Yes | Few small red necrotic spots, no/little chlorotic halo | | No |
|  | Empty | Yes | Few small red necrotic spots, no/little chlorotic halo | | No |
|  | 1 | No | Red necrotic spots with chlorotic halo, spreading lesions 1-10 mm long | | Yes |
|  | 2 | No | Red necrotic spots with chlorotic halo, spreading lesions 1-15 mm long | | Yes |

*(S) = Susceptible genotype, (R) = Resistant genotype
