# Supplementary material for: A highly conserved NB-LRR encoding gene cluster effective against Setosphaeria turcica in sorghum
Source: BMC Plant Biol. 2011 Nov 3;11:151. doi: 10.1186/1471-2229-11-151 (PMC3262770; doi:10.1186/1471-2229-11-151)
Supplement: Additional file 6 — Gene specific primers for VIGS constructs. Restriction sites are in bold. [file 1471-2229-11-151-S6.DOC]

**Additional file 4:** Gene specific primers for VIGS constructs.

| **Construct** | **Forward sequence** | **Reverse sequence** |
| --- | --- | --- |
| 1 | GAGA**CCATGG**GCGGCTTCGAAGTGAGCTA | GAGA**CCTAGG**TACAAGCATATTGCGGTGGA |
| 2 | GAGA**CCATGG**CTGAAGTGCCTGCATCCATA | GAGA**CCTAGG**AGGTCCCAGACATCATCCAA |
