# Supplementary material for: A highly conserved NB-LRR encoding gene cluster effective against Setosphaeria turcica in sorghum
Source: BMC Plant Biol. 2011 Nov 3;11:151. doi: 10.1186/1471-2229-11-151 (PMC3262770; doi:10.1186/1471-2229-11-151)
Supplement: Additional file 7 — List of primers used in real time PCR analysis. [file 1471-2229-11-151-S7.DOC]

**Additional file 5:** List of primers used in real time PCR analysis.

| **Gene** | **Forward sequence** | **Reverse sequence** |
| --- | --- | --- |
| *St1A* | CCAGTGACCTTGTGGAGGTT | AGGGAAGAGGCTGCAGTACA |
| *St1B* | AGGAGGTTGCTGAGGGGAAAT | AAGTGCCGCATTAACATTCC |
| *St2A* | GCACTTGCTCAACCAAGACA | TTGCTGGCAGTGAAGACAAC |
| *St2B* | TTGCTGAAGGATTTGCAGTG | TTATCACTGCAGCCTGATCG |
| *St3A* | GGCACATTCTTGCTGACAGA | CACGCTCCTTAGTTGCATCA |
| *St3B* | **GAAAGAGGATGTGGCTGCTC** | **CCGTCAGACTCGTCGTTGTA** |
| *Sb10g028720* | **GAGACAGATCGAGGGTGAGC** | **TCTTCCACCTCATGGGAGAC** |
| *Sb10g028730* | **GCCTAACTTGAGCAGGTTGC** | **ACACCTGCATCCAACTTTCC** |
| *SbActin* | GCCGTCCTCTCTCTGTATGC | AGGGCGTACCCTTCGTAGAT |
| *SbEF1a* | AGCGTGTCATCGAGAGGTTT | AACTTCCACAGGGCAATGTC |
